# Involvement of Lactate and Pyruvate in the Anti-Inflammatory Effects Exerted by Voluntary Activation of the Sympathetic Nervous System

**DOI:** 10.3390/metabo10040148

**Published:** 2020-04-10

**Authors:** Jelle Zwaag, Rob ter Horst, Ivana Blaženović, Daniel Stoessel, Jacqueline Ratter, Josephine M. Worseck, Nicolas Schauer, Rinke Stienstra, Mihai G. Netea, Dieter Jahn, Peter Pickkers, Matthijs Kox

**Affiliations:** 1 Department of Intensive Care Medicine, Radboud Institute for Molecular Life Sciences, Radboud University Medical Center, Internal Mail 710 Geert Grooteplein 10, 6500HB Nijmegen, The Netherlands; Jelle.zwaag@radboudumc.nl (J.Z.); peter.pickkers@radboudumc.nl (P.P.); 2 Radboud Center for Infectious Diseases (RCI), Radboud University Medical Center, Internal Mail 710 Geert, Grooteplein 10, 6500HB Nijmegen, The Netherlands; RterHorst@cemm.oeaw.ac.at (R.t.H.); Jacqueline.Ratter@radboudumc.nl (J.R.); Rinke.stienstra@radboudumc.nl (R.S.); Mihai.netea@radboudumc.nl (M.G.N.); 3 Department of Internal Medicine, Radboud Institute for Molecular Life Sciences, Radboud University Medical Center, Internal Mail 710 Geert Grooteplein 10, 6500HB Nijmegen, The Netherlands; 4 Institute of Microbiology, Technische Universität Braunschweig, Universitätsplatz 2, 38106 Braunschweig, Germany; ivana.blazenovic@gmail.com (I.B.); d.jahn@tu-bs.de (D.J.); 5 Metabolomic Discoveries, GmbH, Am Mühlenberg 11, 14476 Potsdam, Germany; danielstoessel@yahoo.com (D.S.); josephine@worseck.de (J.M.W.); nschauer@gmail.com (N.S.); 6 Department of Biochemistry and Biology, Universität Potsdam, Am Neuen Palais 10, 14469 Potsdam, Germany; 7 Max Planck Institute für Molekulare Pflanzenphysiologie, Am Mühlenberg 1, 14476 Potsdam, Germany; 8 Nutrition, Metabolism and Genomics Group, Division of Human Nutrition, Wageningen University, Droevendaalsesteeg 4, 6708 PB Wageningen, The Netherlands; 9 Immunology and Metabolism, LIMES Institute, University of Bonn, Carl-Troll-Straße 31, 53115 Bonn, Germany

**Keywords:** metabolomics, LPS, endotoxin, pyruvate, lactate, cytokines, inflammation, human endotoxemia, cori cycle, warburg effect

## Abstract

We recently demonstrated that the sympathetic nervous system can be voluntarily activated following a training program consisting of cold exposure, breathing exercises, and meditation. This resulted in profound attenuation of the systemic inflammatory response elicited by lipopolysaccharide (LPS) administration. Herein, we assessed whether this training program affects the plasma metabolome and if these changes are linked to the immunomodulatory effects observed. A total of 224 metabolites were identified in plasma obtained from 24 healthy male volunteers at six timepoints, of which 98 were significantly altered following LPS administration. Effects of the training program were most prominent shortly after initiation of the acquired breathing exercises but prior to LPS administration, and point towards increased activation of the Cori cycle. Elevated concentrations of lactate and pyruvate in trained individuals correlated with enhanced levels of anti-inflammatory interleukin (IL)-10. In vitro validation experiments revealed that co-incubation with lactate and pyruvate enhances IL-10 production and attenuates the release of pro-inflammatory IL-1β and IL-6 by LPS-stimulated leukocytes. Our results demonstrate that practicing the breathing exercises acquired during the training program results in increased activity of the Cori cycle. Furthermore, this work uncovers an important role of lactate and pyruvate in the anti-inflammatory phenotype observed in trained subjects.

## 1. Introduction

Recent work from our group revealed that the sympathetic nervous system can be voluntarily activated through a training program consisting of exposure to cold, breathing exercises, and meditation [1]. Compared to an untrained control group who did not practice any exercise, a strong increase in endogenous epinephrine levels was observed in trained healthy volunteers shortly after initiation of the learned breathing exercises. This resulted in augmentation of plasma concentrations of interleukin (IL)-10, a pivotal anti-inflammatory cytokine, and suppression of the pro-inflammatory response during experimental human endotoxemia, a standardized and reproducible model of systemic inflammation elicited by lipopolysaccharide (LPS) administration [1]. Activation of the sympathetic nervous system is also known to profoundly impact cellular metabolism [2]. Furthermore, interaction between immune responses and host metabolism, also termed “immunometabolism”, has gained renewed interest, as it was recently discovered that metabolic reprogramming of innate immune cells plays a critical role in mounting effective immune responses and in generation of innate immune memory [3,4]. 

Metabolomic profiling, for instance using reversed phase chromatography coupled to electrospray ionization mass spectrometry (ESI–MS), enables the detection of highly polar compounds in blood [5]. These techniques allow for simultaneous investigation of a large number of metabolites, thereby providing an indication of the “metabolic status” of an individual [6]. Using such an established platform [7], we performed metabolomic profiling of plasma samples obtained in the study described above [1]. Our primary aim was to determine changes in the plasma metabolome of trained subjects [1], and to assess if these changes could play a role in the immunomodulatory effects observed. 

## 2. Results

Characteristics of the study population are listed in Table S1 in the Supplementary Material and reveal no differences between the control group and the trained group. 

### 2.1. Effects of LPS Administration on Plasma Metabolites

**Figure 1:**
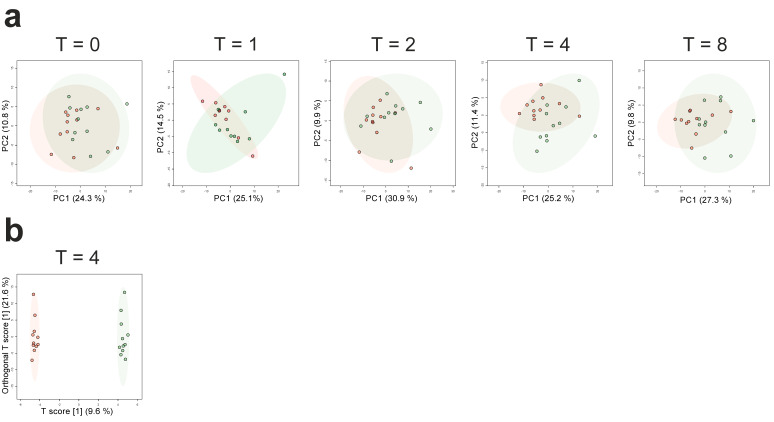
Effects of endotoxemia on the plasma metabolome. (**a**) Principal component analysis (PCA) of the plasma metabolome at T = 0 (just prior to lipopolysaccharide (LPS) administration), 1, 2, 4, and 8 h following LPS administration in the control group. The red dots indicate baseline (one hour before LPS administration) and the green dots are the timepoints, indicated above the graphs. The shaded areas indicate the 95% confidence intervals. (**b**) Orthogonal partial least squares-discriminant analysis (OPLS-DA) of the plasma metabolome at 4 h after LPS administration in the control group. The red dots indicate baseline (one hour before LPS administration) and the green dots indicate 4 h post-LPS administration. The shaded areas indicate the 95% confidence intervals.

We first examined LPS-induced changes on the plasma metabolome. To this end, we restricted the analyses to the control group. Expectedly, LPS administration resulted in typical flu-like symptoms, fever, hemodynamic changes, and increases in plasma concentration of pro- and anti-inflammatory cytokines (results reported in detail elsewhere [1], baseline, peak, and area under curve (AUC) plasma cytokine levels are provided in Table S2 in the Supplementary Material). We compared levels of plasma metabolites 0, 1, 2, 4, and 8 h after LPS administration to those at baseline (one hour before LPS administration). PCA plots are depicted in Figure 1a and show the clearest separation at 4 h post-LPS. 

**Figure 2:**
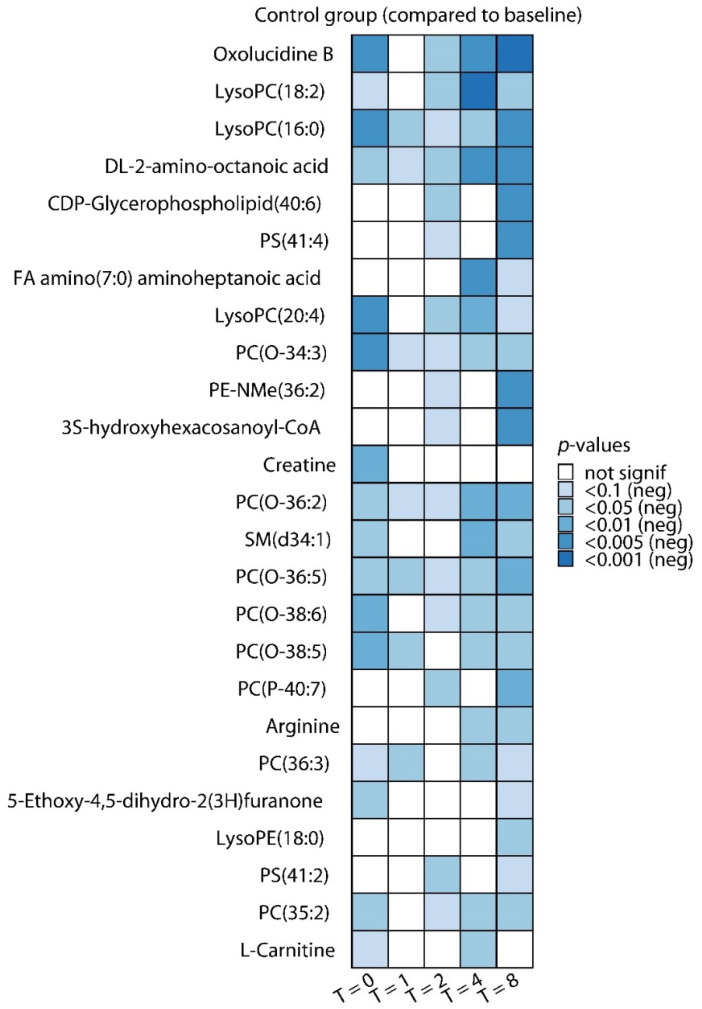
Top differentially regulated metabolites during endotoxemia. Top 25 most significantly (according to *p*-value) regulated metabolites at T = 0 (just before LPS administration), 1, 2, 4, and 8 h post-LPS administration compared with baseline (one hour before LPS administration) in the control group. Blue color indicates a decrease compared with baseline (this top 25 only comprises decreased metabolites). *p*-values were calculated using paired *t*-tests with multiple-testing correction (Benjamini–Hochberg false discovery rate [FDR]).

Subsequently, we constructed orthogonal partial least squares-discriminant analysis (OPLS-DA) models. Permutation tests revealed that only the model constructed for the comparison of T = 4 h versus baseline showed significant separation (1 + 3 components, R^2^X_cum_: 0.448, R^2^Y_cum_: 0.997, Q^2^_cum_: 0.404; permutation tests: R^2^Y: *p* < 0.001, Q^2^: *p* = 0.03, score plot depicted in Figure 1b). Differential analyses revealed 98 significantly altered metabolites out of a total of 224 identified metabolites (false discovery rate [FDR]-adjusted *p*-value of < 0.1) across all timepoints. The top 25 significantly altered metabolites are depicted in Figure 2 (all decreased compared with baseline). A complete overview of all metabolites on all timepoints is provided in Tables S3 and S4 in the Supplementary Material.

The relatively large number of decreased metabolites at T = 0 is likely a result of dilution, as all subjects were pre-hydrated with 1.5 L of saline/glucose solution between baseline and T = 0 as part of our standard experimental endotoxemia protocol [8]. Because PCA and OPLS-DA models showed the most pronounced separation at T = 4 h, we focused on that timepoint, and all 44 significantly altered metabolites (10 increased, 34 decreased) at T = 4 h are listed in Table 1. 

Three of the increased metabolites belonged to the class of nucleosides, nucleotides, and derivatives. The majority of decreased metabolites were comprised of glycerophospholipids. Metabolite Set Enrichment Analysis (MSEA) [9] revealed that the most significantly affected pathways included glutamate metabolism, oxidation of various fatty acids, pyrimidine metabolism, the urea cycle, and the Warburg effect (Figure S1 in the Supplementary Material).

### 2.2. Differences in Plasma Metabolites in Trained and Untrained Individuals 

**Figure 3:**
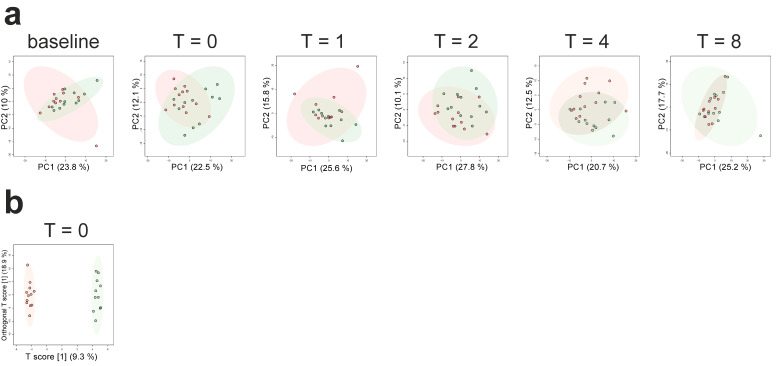
The plasma metabolome in trained and untrained subjects. (**a**) Principal component analysis (PCA) of the plasma metabolome at baseline (one hour before LPS administration, T = 0 (just before LPS administration), and 1, 2, 4, and 8 h post-LPS administration in the control (red dots) and trained (green dots) group. The shaded areas indicate the 95% confidence intervals. (**b**) Orthogonal partial least squares-discriminant analysis (OPLS-DA) of the plasma metabolome at T = 0 in the control (red dots) and trained (green dots) group. The shaded areas indicate the 95% confidence intervals.

We performed PCA and constructed OPLS-DA models for the control versus trained group at each timepoint. PCA plots did not show clear separation (Figure 3a). 

**Figure 4:**
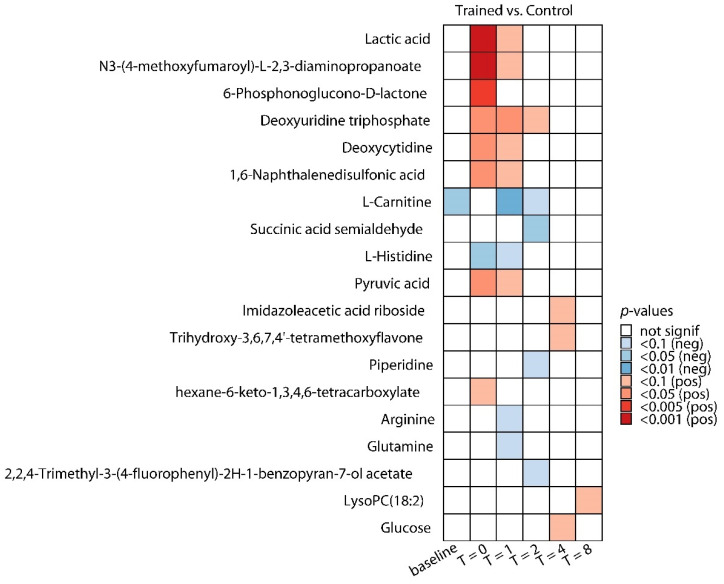
Differentially regulated metabolites in the trained group. Significantly regulated metabolites at baseline (one hour before LPS administration), T = 0 (immediately prior to LPS administration), and 1, 2, 4, and 8 h following LPS administration in the training group compared with the control group. Blue color indicates a decrease compared with the control group, red color indicates an increase compared with the control group. *p*-values were calculated using unpaired *t*-tests with multiple-testing correction (Benjamini–Hochberg false discovery rate [FDR]).

Permutation tests revealed that the model constructed for the comparison between both groups at T = 0 h (when the trained individuals had been practicing the breathing exercises for 30 min but LPS had not yet been administered) showed significant separation (1 + 3 components, R^2^X_cum_: 0.439, R^2^Y_cum_: 0.995, Q^2^_cum_: 0.649; permutation tests: R^2^Y: *p* = 0.03, Q^2^: *p* = 0.001, score plot depicted in Figure 3b), whereas models constructed at other timepoints did not. Differential analyses revealed a total of 19 significantly altered metabolites between trained and untrained groups across all timepoints (Figure 4). 

At baseline (after the trained subjects had completed the training program but before any endotoxemia-related procedures or breathing exercises were performed), L-carnitine was lower in the trained group. Nevertheless, the vast majority of significant between-group changes were observed at timepoints between T = 0 and T = 2 h, during which the trained group practiced the breathing exercises acquired during their training program. A list of all metabolites across all timepoints in both groups is provided in Tables S3 and S4 in the Supplementary Material. Based on the OPLS-DA model that showed significant separation between both groups at T = 0, we focused on that particular timepoint, and all 9 significantly altered metabolites (8 up, 1 down) at T = 0 are listed in Table 2. 

**Figure 5:**
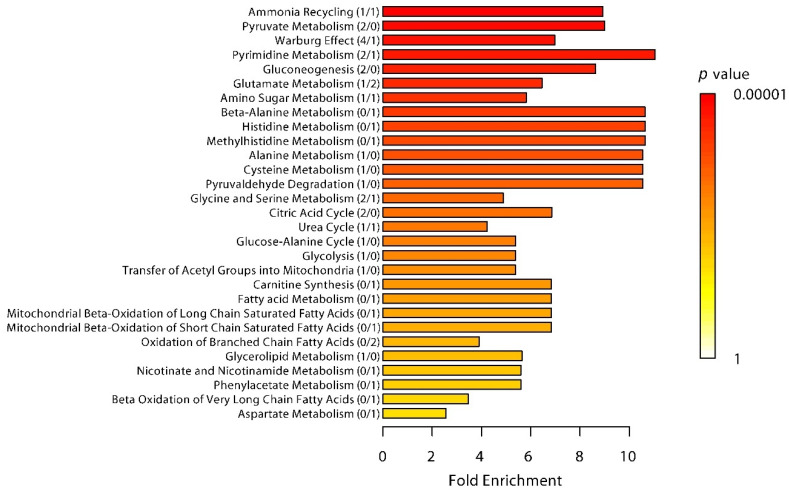
Enriched pathways in the trained group. Significantly enriched pathways at T = 0 in the trained group compared with the control group. Threshold for significance was set at a Benjamini–Hochberg false discovery rate [FDR]-adjusted *p*-value of less than 0.1. Number of significantly increased/decreased metabolites within each pathway are indicated in parentheses.

Lactic acid (lactate) was one of the most significantly increased metabolites in the trained group. We previously measured this metabolite at the bedside using a point-of-care blood analyzer (i-STAT) [1], which provided us with an opportunity to validate the liquid chromatography-mass spectrometry (LC-MS) analysis for this metabolite. Lactate concentration measurements were virtually identical between the two methods in both groups (Figure S2A,B in the Supplementary Material). Furthermore, there was excellent correlation between both methods, with only a single outlier sample observed (r values of 0.90 and 0.97 with and without the outlier sample in the analysis, respectively, Figure S2C in the Supplementary Material). Two other metabolites that were increased (deoxyuridine triphosphate and deoxycytidine) belong to the nucleosides, nucleotides, and derivates class, while 6-phosphonoglucono-d-lactone is an intermediate in the pentose phosphate pathway, and pyruvic acid (pyruvate) is a well-known metabolite of the alcohols and polyols class. The only decreased metabolite was l-histidine, an amino acid used in the biosynthesis of proteins. MSEA enrichment analysis revealed that the most significantly affected pathways included ammonia recycling, pyruvate metabolism, the Warburg effect, pyrimidine metabolism, gluconeogenesis, glutamate metabolism, and amino sugar metabolism (Figure 5).

### 2.3. Relationship between Differentially Regulated Metabolites and Inflammatory Response Mediators

**Figure 6:**
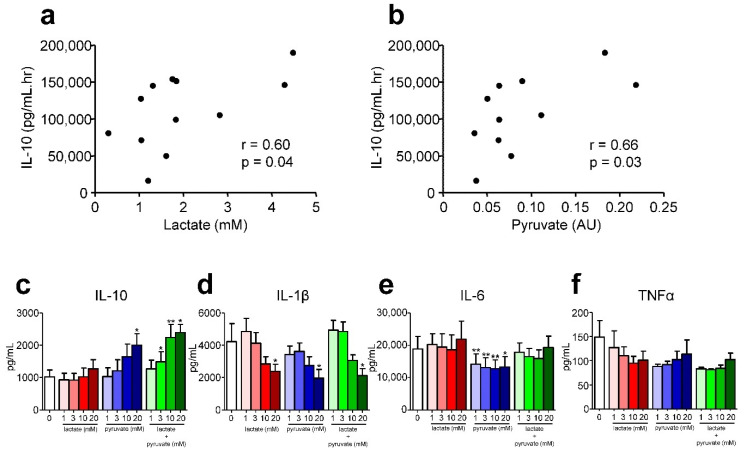
Role of lactate and pyruvate in interleukin (IL)-10 induction. (**a**–**b**) Relationship between plasma lactate (**a**) and pyruvate (**b**) levels at T = 1 and the plasma IL-10 response (expressed as area under the time-concentration curve (AUC)). (**c**–**f**) IL-10, IL-1β, IL-6 and tumor necrosis factor (TNF)α production by LPS-stimulated peripheral blood mononuclear cells (PBMCs) incubated with medium alone (white bar) or different concentrations of lactate (red bars), pyruvate (blue bars), or a combination of the two (green bars). Spearman correlation was used to calculate r and *p*-values in panels a and b. Data in panels c–f are depicted as mean ± standard error of the mean (SEM) of 6 (panels c–e) or 3 (panel f) donors.* *p* < 0.05, ** *p* < 0.01 versus medium control (paired *t*-tests).

We evaluated whether in trained individuals practicing the breathing exercises, plasma concentrations of the significantly altered metabolites listed in Table 2 at early timepoints correlated with in vivo cytokine responses, which were profoundly modulated in these subjects (i.e., strongly increased plasma levels of the anti-inflammatory cytokine IL-10 and attenuated concentrations of pro-inflammatory cytokines tumor necrosis factor (TNF)α, IL-6, and IL-8, see Table S2 in the Supplementary Material and Reference [1]). At T = 0, only one significant correlation was found, between l-histidine and IL-8 (r = 0.62, *p* = 0.048). At T = 1, significant correlations between several metabolites and plasma levels of IL-10, which were enhanced by three-fold in trained subjects compared with the control group (Table S2 in the Supplementary Material and Reference [1]), were found. These include 1,6-naphthalenedisulfonic acid (r = 0.76, *p* = 0.02), deoxyuridine triphosphate (r = 0.78, *p* = 0.03), and deoxycytidine (r = 0.74, *p*=0.03). Interestingly, also lactate and pyruvate levels at T = 1 correlated with the AUC IL-10 response (r = 0.60, *p* = 0.04 and r = 0.66, *p* = 0.03, respectively, Figure 6a,b). 

Furthermore, these two metabolites were highly intercorrelated (r = 0.94, *p* < 0.0001), but were not related to the profoundly increased epinephrine levels observed in trained subjects practicing the breathing exercises at any of the measured timepoints (*p*-values > 0.15), which were previously shown to be an important driver of enhanced IL-10 production [1]. Because both lactate and pyruvate have been implicated to exert anti-inflammatory effects [10,11], we set out to validate the observed associations using in vitro experiments with peripheral blood mononuclear cells (PBMCs) obtained from healthy individuals. High concentrations of pyruvate, but not lactate, enhanced LPS-induced IL-10 production, and the combination of both metabolites resulted in an even more pronounced and statistically robust increase (Figure 6c). Furthermore, pyruvate attenuated production of the pro-inflammatory cytokines IL-1β and IL-6 (Figure 6d,e), whereas high concentrations of lactate as well as the combination of both metabolites mitigated IL-1β production (Figure 6d). LPS-induced TNFα production was rather low, it was detectable in cell culture supernatants of only three out of the six healthy donors (data of these subjects are shown in Figure 6f). This is likely due to the fact that the 48 h incubation time is too prolonged to reliably measure TNFα responses, as levels in supernatants were previously shown to decline as early as 6 h following LPS stimulation [12]. Nevertheless, lactate, pyruvate, and the two metabolites combined also tended to attenuate LPS-induced TNFα production, although significance was not reached, probably due to the low remaining sample size.

## 3. Discussion

In the present work, we demonstrated that the systemic inflammatory response induced by LPS in healthy volunteers significantly alters the plasma metabolome, with the most profound changes taking place 4 h following LPS administration. Endotoxemia mainly led to an increase of several nucleosides, nucleotides, and derivatives, and a decrease in many glycerophospholipids. Before LPS administration, trained subjects practicing the learned breathing exercises exhibited higher levels of lactate and pyruvate compared with a control group who did not practice any exercise, and concentrations of these metabolites correlated with the profoundly enhanced levels of the anti-inflammatory cytokine IL-10 observed in trained individuals following LPS administration. We subsequently validated these findings in vitro, by showing that co-incubation with lactate and pyruvate enhances LPS-induced IL-10 release and attenuates pro-inflammatory cytokine production by primary human leukocytes.

Two of the most profoundly enhanced metabolites after the LPS challenge were deoxyuridine triphosphate and especially, deoxycytidine, both involved in the pyrimidine metabolism pathway, which was enriched following LPS administration. Increased plasma deoxycytidine levels result from DNA degradation [13]. We have previously shown that plasma levels of both nuclear and mitochondrial DNA show a transient increase during endotoxemia, reaching their maximum levels 3 h after LPS administration, after which they gradually return to baseline in the following hours [14]. Hence, it might be speculated that the breakdown of plasma DNA is one of the main drivers of the increased deoxycytidine concentrations observed 4 h after LPS administration. Many metabolites belonging to the class of glycerophospholipids were distinctly decreased following administration of LPS. Related to this, various pathways related to lipid metabolism were enriched, which is in accordance with previous findings obtained in healthy volunteers undergoing experimental endotoxemia [15]. Glycerophospholipids are the main components of cell membranes and function as precursors to signaling molecules involved in many cellular and physiological processes [16]. The majority of the decreased metabolites belonging to this class consisted of phosphatidylcholines. Previous work from our group has shown that plasma levels of secretory phospholipase A2 (sPLA_2_), the principal catalysts of glycerophospholipid hydrolysis, greatly increase during endotoxemia [17], which may be an explanation for the reduced plasma glycerophospholipid concentrations. The Warburg effect is another pathway of interest that was enriched during systemic inflammation, with four significantly increased (6-phosphonoglucono-D-lactone, glucose, lactate, pyruvate) and one significantly decreased (l-glutamine) metabolite. This process entails the shift from oxidative phosphorylation (OXPHOS) as the primary energy source towards aerobic glycolysis and was recently shown to play a critical role in mounting (LPS-induced) immune responses, by, among other things, facilitating rapid production of inflammatory cytokines [18,19,20]. 

Following the training program, trained subjects exhibited lower plasma levels of L-carnitine compared with untrained controls. This relative depletion of carnitine from plasma may indicate an overall increment in Acyl-shuttling mechanism in the mitochondria by CPT1/CPT2 and might be related with lipid beta-oxidation intensification [21]. The most pronounced changes in the plasma metabolome of trained subjects compared with controls were, however, observed during the period in which the breathing exercises were carried out. This was not unexpected given the major changes in cardiorespiratory parameters, markers of autonomic nervous system activity, and inflammatory molecules and symptoms observed during this period, as reported elsewhere [1]. At T = 0, when the trained individuals had been practicing the breathing exercises for 30 min but just prior to LPS administration, the Warburg effect was one of the top enriched pathways in trained individuals compared with controls. Plasma levels of four metabolites (lactate, 6-phosphonoglucono-D-lactone, pyruvate, and isocitric acid) of this pathway were increased in the trained group, whereas concentrations of l-glutamine were lower. This finding may be counterintuitive, as the Warburg effect is mainly associated with pro-inflammatory effects, whereas trained subjects exhibited a distinct anti-inflammatory phenotype [1]. The fact that LPS, a strong inducer of the Warburg effect [20], had not yet been administered at this early timepoint, renders this finding biologically implausible as well. Finally, as alluded to before, the Warburg effect encompasses a shift from oxidative phosphorylation (and thus usage of the citric acid cycle) towards glycolysis, but the citric acid cycle pathway was also enhanced in the trained group. Therefore, we hypothesize that the observed effect is not due to a true Warburg effect (*aerobic* glycolysis), but rather driven by the profoundly increased lactate levels in this group as a result of classical *anaerobic* glycolysis in the muscles. The latter may have been caused by the combination of vigorous breathing (including repeated muscle tightening), intermittent hypoxia, and epinephrine-induced vasoconstriction in this group [1]. The breathing exercises could certainly be regarded as exercise, given that, similar to exercise, they resulted in profuse sweating and exhaustion. The fact that lactate and pyruvate levels were highly intercorrelated provides a strong indication of increased gluconeogenesis (i.e., the generation of glucose from non-carbohydrate substrates such as lactate, with pyruvate as the intermediate metabolite, which mainly takes place in the liver). Gluconeogenesis was also one of the top enhanced pathways in the trained group during practicing of the breathing exercises. Because epinephrine is a well-known strong inducer of gluconeogenesis [22], the profoundly increased plasma epinephrine levels observed shortly after initiation of the breathing exercises [1] likely play a pivotal role in this effect. Combining the findings of enhanced anaerobic glycolysis with increased gluconeogenesis, our data provide evidence of increased activation of the Cori cycle in trained subjects during practicing of the breathing exercises, in which lactate is produced by muscle cells and subsequently converted back, via pyruvate, into glucose in the liver [23]. However, it needs to be acknowledged that we can only infer activation of the Cori cycle, as we did not perform isotype labeling experiments. 

Correlation analysis between differentially regulated metabolites and inflammatory cytokine levels in trained subjects revealed that both lactate and pyruvate were related to plasma concentrations of the key anti-inflammatory cytokine IL-10. Of note, production of this cytokine was profoundly accelerated and enhanced in trained subjects practicing the breathing exercises, and its plasma levels were strongly correlated to the subsequent attenuation of pro-inflammatory cytokine responses [1]. Our PBMC stimulation experiments demonstrated that pyruvate alone, but especially the combination of both pyruvate and lactate, exert a robust enhancing effect on LPS-induced IL-10 production. These results may substantiate the correlations found during endotoxemia in vivo. Furthermore, both lactate and pyruvate individually, as well as the combination of both metabolites, attenuate IL-1β production, and lactate mitigated the release of IL-6, another pro-inflammatory cytokine. Both lactate and pyruvate have been demonstrated previously to exert anti-inflammatory effects. For instance, very recent work demonstrates that addition of lactate to LPS-stimulated primary human monocytes causes a distinct metabolic shift by decreasing aerobic glycolysis and increasing oxidative phosphorylation, a metabolic state characteristic for anti-inflammatory responses [24]. In the same study, lactate was shown to decrease LPS-induced production of pro-inflammatory cytokines by human PBMCs [24]. The authors proposed that immunomodulatory effects of lactate may serve as a feedback signal to limit excessive inflammatory responses of highly glycolytic pro-inflammatory immune cells [24]. Pyruvate has been shown to decrease mRNA expression and protein levels of pro-inflammatory cytokines TNFα and IL-6 in LPS-stimulated canine PBMCs, whereas IL-10 expression and production was increased [25]. Furthermore, administration of pyruvate significantly lowered IL-6 and enhanced IL-10 plasma concentrations in LPS-treated rats, leading to prolonged survival, and incubation with ethyl pyruvate blocked activation of nuclear factor (NF)-κB, a critical pro-inflammatory transcription factor, in LPS-stimulated murine macrophages [26]. These data strengthen the notion that also in the human in vivo situation, the observed increases in lactate and pyruvate at least partly account for the immunomodulatory effects observed in trained subjects. 

Several limitations and aspects of the present work deserve attention. First, only male volunteers were included. In earlier work, we observed that the endotoxin-induced pro-inflammatory immune response is more pronounced in females than in males [27]. Furthermore, menstrual cycle-induced variation in hormone levels can also impact immune parameters [28,29], thereby further increasing variation. Therefore, inclusion of both sexes yields more inter-individual variation and would necessitate larger group sizes. Because endotoxemia studies are very labor-intensive and costly, the choice was made to only include males, which nevertheless limits this study’s generalisability.

A second limitation, which applies to all plasma metabolomics studies, pertains to the uncertainty of the source of the measured metabolites. Virtually all identified metabolites can be produced by a wide variety of cells in many organs. Especially in immunological studies like this, combining plasma metabolomics with the determination of intracellular metabolites in immune cells (e.g., leukocytes) would represent a more powerful approach. Unfortunately, no samples were stored for this purpose. 

Third, the current study does not allow us to deduce which (combination) of the three elements of the training program is responsible for the observed effects, and several of our ongoing studies are aimed at elucidating this question. Nevertheless, as alluded to in a previous section, the breathing exercises probably play a pivotal role. 

Fourth, because metabolomic profiling was performed on samples stored for three years at −80 °C, sample degradation could be an issue. Nevertheless, the high correlation between lactate measured during the experiments using a point-of-care analyzer and by LC-MS indicates that, at least for this metabolite, no significant degradation occurred during storage. Furthermore, because storage time was virtually identical across samples (all were collected within one month), the extent of degradation of certain metabolites would have been similar across all samples. Provided that metabolites did not completely degrade, this would therefore have little impact on the results.

Fifth, as the PBMCs stimulation experiments with lactate and pyruvate contained a mixture of different cell types (predominantly monocytes and lymphocytes), we cannot be certain about the cellular origin of the effects observed. However, it is well established that monocytes are the main cytokine producers in short-term whole blood LPS stimulation assays (which next to PBMCs also contain granulocytes) [30]. Furthermore, as discussed earlier on, anti-inflammatory effects of lactate and pyruvate have been reported in primary human monocytes [24] and murine macrophages [26], the latter of which show many similarities to monocytes. Therefore, it is likely that the effects of pyruvate and lactate we observed can predominantly be ascribed to monocytes. 

Finally, and importantly, all training procedures described in this study were conducted in the presence of medical personnel. Because of profound physiological effects of the breathing exercises (e.g., acid-base shifts, intermittent hypoxia) and exposure to cold [1], potential health risks while practicing these elements of the training program should be considered.

In conclusion, the present study extends our previous findings regarding the effect of a training intervention consisting of cold exposure, breathing exercises, and meditation on the LPS-induced immune response in healthy volunteers. Practicing the breathing exercises acquired during the training program results in enhanced activity of the Cori cycle, and next to the previously established relationship between epinephrine and IL-10 induction [1], the current data indicate a role of lactate and pyruvate in the enhanced production of this key anti-inflammatory mediator and in the overall anti-inflammatory phenotype observed in trained subjects.

## 4. Materials and Methods 

### 4.1. Subjects and Experimental Design

Metabolomic profiling was performed in plasma samples obtained in a previously published parallel randomized controlled study registered at ClinicalTrials.gov as NCT01835457 [1]. The study protocol is described in detail elsewhere [1]. Briefly, after approval from the local ethics committee of the Radboud University Medical Center (CMO 2012/455), 24 healthy nonsmoking male volunteers with a median age of 22 years (range 19–27) provided written informed consent and were included in the study. All study procedures were in accordance with the Declaration of Helsinki, including current revisions, and Good Clinical Practice guidelines. Subjects were screened before the study and had a normal physical examination, electrocardiography, and routine laboratory values. Exclusion criteria were: febrile illness during the 2 weeks before the endotoxemia experiment, taking any prescription medication, history of spontaneous vagal collapse, practicing or experience with any kind of meditation, or participation in a previous trial where LPS was administered. The subjects were randomly allocated to the trained group (n = 12) or the control group (n = 12) by the opening of sealed envelopes prepared by unblinded staff not involved in the study. The trained group underwent a 10 day training program provided by Dutch individual Wim Hof, which consisted of three main elements: meditation, exposure to cold, and breathing exercises (see Reference [1] for a detailed description). After completion of the training, subjects of both groups (n = 24) underwent experimental human endotoxemia at our intensive care research unit, consisting of administration of an intravenous bolus of 2 ng/kg of US Standard Reference *Escherichia coli* endotoxin (*E. coli* O:113 (LPS), Clinical Center Reference Endotoxin; National Institutes of Health, Bethesda, MD, USA). As part of our standard endotoxemia protocol [31,32], the subjects received 1.5 L 0.9% NaCl over one hour, starting one hour before endotoxin infusion (pre-hydration), followed by 150 mL/h until 6 h after endotoxin infusion and 75 mL/h until the end of the experiment. The experimental human endotoxemia protocol is provided in detail elsewhere [1,32]. The control group did not undergo any training procedures and also underwent experimental human endotoxemia. Subjects in the trained group started practicing the breathing exercises acquired during the training program 30 min before LPS administration until two-and-a-half h afterwards. The control group did not practice any exercise throughout the endotoxemia experiment. As described in detail elsewhere [1], the breathing exercises consisted of two exercises. In brief, the first exercise comprised cycles of vigorous hyperventilation (approximately 30 breaths) followed by breath holding for several minutes at the discretion of the subject. The second exercise was similar, but at the end of the hyperventilation period, breath was only held for 10 s during which all body muscles were tightened. 

### 4.2. Sample Preparation for LC-MS Analysis

Ethylenediaminetetraacetic acid (EDTA) anti-coagulated blood was obtained one hour before LPS administration (baseline), and at T = 0, T = 1, T = 2, T = 4, and T = 8 h. Blood was immediately centrifuged at 2000 *g* for 10 min at 4 °C, after which plasma was stored at −80 °C until analysis. Sample extraction was performed as described previously [7], with some modifications. Briefly, 50 µL of plasma was mixed with 450 µL of 90% (*v*/*v*) methanol containing internal standards and incubated for 15 min at 37 °C with 1150 rpm. Precipitated proteins were separated from the extract by centrifugation for 12 min at 15,000 rpm, after which supernatants were stored at −80 °C until further analysis.

### 4.3. LC-MS Analysis 

Modified reversed-phase chromatography in combination with high resolution mass spectrometry (HRMS) was employed in this study. Samples were analyzed on an Agilent 1290 ultra high performance liquid chromatography (UPLC) system (Agilent) with a Discovery HS F5-3 column (15 cm × 2.1 mm, 3 µm, Supelco, Sigma Aldrich) coupled to a high-resolution 6540 quadrupole time-of-flight mass spectrometry (QTOF/MS) detector (Agilent) operated in both positive and negative electrospray ionization (ESI) mode in a detection range of 50 to 1700 *m/z* at 2 GHz in extended dynamic range. The liquid chromatography (LC) solvent consisted of 95% 10 mM ammonium formate with 0.1% formic acid and 5% acetonitrile (A), and 95% acetonitrile with 5% 10 mM ammonium formate with 0.1% formic acid (B). A multi-step gradient was used, with 5% B from 0–0.1min to 35% B at 1.5 min, to 95% B at 2.05 min which was kept constant until 3.2 min, to 5% B at 3.21 min and washing until 4.3 min with 5% B. The flow rate was kept constant at 700 µL/min from 0 to 2.2 min, and increased up to 900 µL/min from 2.2 to 2.5 min, after which flow rate was kept constant until 3.2 min. The flow rate was decreased from 900 to 800 µL/min from 3.2 to 3.1 min and kept constant until 3.7 min, when the flow rate was changed to 700 µL/min. The run time was 4.3 min, 1 µl of sample was injected and the column heated to 40 °C. The DualAJS ESI source was set to the following parameters: Gas temperature 200 °C, drying gas 8 L/min, nebulizer 35 pounds-force per square inch gauge (psig), sheath gas temp: 350 °C, sheath gas flow 11 L/min, capillary voltage (VCap) 3500 V and nozzle voltage of 0 V. Online calibration of the instrument was performed throughout the data acquisition using Agilent ES-TOF Reference Mass Solution Kit.

### 4.4. Cytokine and Lactate Determinations

Plasma concentrations of the cytokines TNFα, IL-6, IL-8, and IL-10 at various timepoints during the endotoxemia experiment were determined by Luminex assay (Milliplex, Millipore), as described previously [1]. The area under the cytokine plasma concentration-time curves (AUC) was used as an integral measure of the subjects’ in vivo cytokine responses. Furthermore, we validated the LC-MS analysis for lactate by comparing LC-MS data with lactate concentrations measured by a point-of-care blood analyzer (i-STAT, Abbot), as described previously [1]. 

### 4.5. PBMC Stimulation Experiments

After approval from the local ethics committee of the Radboud university medical center (CMO 2010/10), EDTA anticoagulated blood was obtained from 6 healthy donors. Isolation of PBMCs was performed by differential centrifugation over Ficoll-Paque PLUS (GE Healthcare Biosciences) in SepMate tubes (STEMCELL technologies). PBMCs were washed thrice with phosphate buffered saline (PBS), counted, and 5 × 10^5^ PBMCs/well were seeded in 96-well round-bottom plates in Roswell Park Memorial Institute (RPMI) 1640 culture medium (Dutch Modification, Invitrogen) supplemented with 50 μg/mL gentamycin (Thermo Fisher Scientific) and 2 mM Glutamax (Invitrogen). Cells were incubated with RPMI (control), or 1, 3, 10, or 20 mM of sodium lactate (provided by the Department of Pharmacy, Radboud University Medical Center, Nijmegen, The Netherlands), pyruvate (Invitrogen), or a combination of sodium lactate and pyruvate for 1 h at 37 °C and 5% CO_2_. Subsequently, RPMI or 10 ng/mL *E. coli*-derived LPS (serotype O55:B5, Sigma Aldrich) was added and PBMCs were incubated for 48 h at 37 °C and 5% CO_2_. Concentrations of IL-10, IL-1β, IL-6, and TNFα in the cell culture supernatants were measured by enzyme-linked immunosorbent assay (ELISA, R&D systems).

### 4.6. Raw Data Processing and Statistical Analyses

Chromatograms were generated by the LC-MS instrument in .d format. Raw data were converted into mzXML and chromatogram peaks were extracted using XCMS v1.42.0 [33], which was optimized using the IPO R package [34] with the following settings: peakwidth = c (10, 70), ppm = 20, snthresh = 10, mzdiff = 0.0034, prefilter = c (3, 100), noise = 100, gapInit = 0.8448, gapExtend = 2.0544, bw = 5, mzwid = 0.015, minfrac = 0.5, max = 50. All further analyses were performed in R programming language [35], Metaboanalyst 4.0 [9], and GraphPad Prism version 5.0 (GraphPad Software). IDEOM software (http://mzmatch.sourceforge.net/ideom.php) [36] was used to eliminate noise and for putative peak annotation by exact mass within ±10 ppm against the Metabolomic Discoveries in the house metabolite library [37] in negative and positive ESI mode, respectively. Retention time prediction was applied to aid metabolite annotation. 972 peaks were detected. Non-annotated metabolites and potential peptides were removed (n = 748), leaving 224 metabolites in the dataset. Furthermore, 6 outlier samples were identified using ROBPCA by defining the sample distances within the orthogonal to the projection plane [38]. This left a total of 138 samples to be analyzed. Principal component analysis (PCA) and orthogonal partial least squares-discriminant analysis (OPLS-DA) were performed to visualize the metabolic alterations between samples after log-transformations followed by mean-centering and dividing by the standard deviation of each variable. OPLS-DA models were validated by permutation tests (1000 permutations) [39]. Differential analyses were performed using paired (within-group comparisons) and unpaired (between-group comparisons) *t*-tests with multiple-testing correction using Benjamini–Hochberg false discovery rate (FDR) [40]. Analogous to previous work [15,41], a FDR-adjusted *p*-value of 0.1 was set as a threshold for statistical significance in the differential analyses. Targeted analyses (permutation tests, Spearman correlation between metabolites and cytokines, and paired *t*-tests for the PBMC stimulation data) were performed without applying the FDR correction, and the threshold for significance was set at *p* < 0.05 for these data. We restricted correlation analyses to metabolite levels at early timepoints (T = 0 and T = 1), because these can still relevantly affect the immunological response, which is orchestrated in the first hour and peaks 1.5 to 2 h after LPS administration [1]. 

The metabolomics dataset is available at the National Institutes of Health (NIH) Common Fund’s National Metabolomics Data Repository (NMDR) website, the Metabolomics Workbench, https://www.metabolomicsworkbench.org, where it has been assigned Project ID PR00083. The data can be accessed directly via it’s Project DOI: 10.21228/M8C671. Metabolomics Workbench is supported by NIH grant U2C-DK119886.

## Figures and Tables

**Table 1:** Significantly altered metabolites 4 h after LPS administration in the control group.

Metabolite	Class	Fold Change Versus Baseline ^1^	FDR Adjusted *p*-Value ^2^
*Increased*			
5,8-Dihydro-6-(4-methyl-3-pentenyl)-1,2,3,4-tetrathiocin	Prenol lipids	+1.25	0.044
6-Phosphonoglucono-D-lactone	Monosaccharides	+3.31	0.051
Deoxycytidine	Nucleosides, nucleotides, and derivatives	+24.77	0.061
Artonin K	Flavonoids	+1.43	0.065
Hexane-6-keto-1,3,4,6-tetracarboxylate	Unknown	+3.91	0.065
Cis-(homo)2aconitate	Metabolism of cofactors and vitamins	+1.25	0.071
Deoxyuridine triphosphate	Nucleosides, nucleotides, and derivatives	+4.46	0.076
Imidazoleacetic acid riboside	Nucleosides, nucleotides, and derivatives	+1.31	0.076
Lactic acid	Hydroxy acids and derivatives	+2.03	0.091
Gnidicin	Unknown	+6.87	0.096
*Decreased*			
LysoPC(18:2)	Glycerophospholipids	−2.22	0.0008
dl-2-amino-octanoic acid	Amino acids and derivatives	−1.51	0.002
Oxolucidine B	Unknown	−2.27	0.005
FA amino(7:0) aminoheptanoic acid	Fatty acyls	−1.59	0.005
LysoPC(20:4)	Glycerophospholipids	−2.85	0.007
PC(O−36:2)	Glycerophospholipids	−1.26	0.007
SM(d34:1)	Sphingolipids	−1.21	0.009
Arginine	Amino acids and derivatives	−1.40	0.013
PC(36:3)	Glycerophospholipids	−1.29	0.015
PC(O-36:5)	Glycerophospholipids	−1.24	0.019
PC(O-34:3)	Glycerophospholipids	−1.28	0.021
l-Carnitine	Alkylamines	−1.32	0.022
Succinic acid semialdehyde	Fatty acids and conjugates	−1.47	0.022
PC(O-38:5)	Glycerophospholipids	−1.20	0.023
PS(21:0)	Glycerophospholipids	−3.43	0.024
l-Acetylcarnitine	Fatty acid esters	−1.67	0.026
PC(O-38:6)	Glycerophospholipids	−1.21	0.027
Glutamine	Amino acids and derivatives	−1.45	0.028
PC(36:5)	Glycerophospholipids	−1.65	0.035
PC(36:4)	Glycerophospholipids	−1.20	0.035
LysoPC(16:0)	Glycerophospholipids	−2.20	0.038
PC(P-40:6)	Glycerophospholipids	−1.19	0.045
PC(35:2)	Organic phosphoric acids and derivatives	−1.25	0.045
Narciclasine	Unknown	−1.86	0.045
PC(40:6)	Glycerophospholipids	−1.69	0.045
Lenticin	Unknown	−1.35	0.065
PC(36:2)	Glycerophospholipids	−1.35	0.066
PC(34:3)	Glycerophospholipids	−1.23	0.071
PC(38:4)	Glycerophospholipids	−1.20	0.071
PE(39:1)	Glycerophospholipids	−1.33	0.071
PC(O-34:2)	Glycerophospholipids	−1.30	0.071
SM(d34:2)	Sphingolipids	−1.20	0.078
PC(38:5)	Glycerophospholipids	−1.17	0.087
TG(41:0)	Glycerolipids	−1.20	0.091

^1^ Baseline represents one hour before LPS administration. ^2^ *p*-values were calculated using paired *t*-tests with multiple-testing correction (Benjamini–Hochberg false discovery rate [FDR]).

**Table 2:** Significantly altered metabolites between the trained and control group at T = 0 (30 min after initiation of the breathing exercises and prior to LPS administration).

Metabolite	Class	Fold-Change (Trained/Control)	FDR-Adjusted *p*-Value ^1^
*Increased*			
N3-(4-methoxyfumaroyl)-l-2,3-diaminopropanoate	Unknown	+3.15	0.0007
Lactic acid	Carbohydrate metabolism	+3.00	0.0007
6-Phosphonoglucono-d-lactone	Lactones	+3.19	0.005
Deoxyuridine triphosphate	Nucleosides, nucleotides, and derivatives	+4.91	0.006
Deoxycytidine	Nucleosides, nucleotides, and derivatives	+9.15	0.007
1,6-Naphthalenedisulfonic acid	Unknown	+2.19	0.007
Pyruvic acid	Alcohols and polyols	+2.07	0.035
Hexane-6-keto-1,3,4,6-tetracarboxylate	Unknown	+3.52	0.083
*Decreased*			
l-Histidine	Amino acids and derivatives	−2.72	0.026

^1^*p*-values were calculated using unpaired *t*-tests with multiple-testing correction (Benjamini–Hochberg false discovery rate [FDR]).

## Supplementary Materials

The following are available online at https://www.mdpi.com/2218-1989/10/4/148/s1: Figure S1: Enriched pathways after LPS administration, Figure S2: Comparison of plasma lactate concentrations measured by LC-MS and a point-of-care analyzer. Table S1: Subject characteristics, Table S2: Baseline, peak, and area under curve (AUC) plasma cytokine responses in control and trained groups, Table S3: Changes in plasma metabolite levels during endotoxemia in the control group. Table S4: Changes in plasma metabolite levels during endotoxemia in the trained group.
