# Supplementary material for: Involvement of Lactate and Pyruvate in the Anti-Inflammatory Effects Exerted by Voluntary Activation of the Sympathetic Nervous System
Source: Metabolites. 2020 Apr 10;10(4):148. doi: 10.3390/metabo10040148 (PMC7652234; doi:10.3390/metabo10040148)
Supplement: Supplementary file 1 [file metabolites-10-00148-s001.zip › Tables S1-S2 and Figures S1-S2.docx]

**Supplementary Material**

**Manuscript title:** Involvement of lactate and pyruvate in the anti-inflammatory effects exerted by voluntary activation of the sympathetic nervous system

**Authors:** Jelle Zwaag, Rob ter Horst, Ivana Blaženović, Daniel Stoessel, Jacqueline Ratter, Josephine M Worseck, Nicolas Schauer, Rinke Stienstra, Mihai G. Netea,
Dieter Jahn, Peter Pickkers, Matthijs Kox

**Table S1**: Subject characteristics.

| **Parameter** | **Control group (n = 12)** | **Trained group (n = 12)** | ***p*-value** |
| --- | --- | --- | --- |
| Age, yrs | 22 (19-27) | 24 (19-27) | 0.43 |
| Height, cm | 185 (179-189) | 181 (172-190) | 0.30 |
| Weight, kg | 78 (65-91) | 75 (58-92) | 0.25 |
| BMI, kg/m^2^ | 23 (20-27) | 23 (19-26) | 0.98 |
| HR, beats/min | 61 (40-75) | 60 (41-80) | 0.88 |
| MAP, mmHg | 94 (78-105) | 92 (82-113) | 0.89 |

Parameters were measured during the screening visit (so before start of the training in the trained group). BMI: body mass index; HR: heart rate; MAP: mean arterial blood pressure. Data are presented as median (range).
*p*-values were calculated using Mann-Whitney U-tests. These data were published previously [1].

**Table S2**: Baseline, peak, and area under curve (AUC) plasma cytokine responses in control and trained groups.

| **Parameter** | **Baseline (pg/mL)** | | **Peak (pg/mL)** | | **AUC (x10^3^ pg/mL.h)** | |
| --- | --- | --- | --- | --- | --- | --- |
|  | **Control** | **Trained** | **Control** | **Trained** | **Control** | **Trained** |
| **TNF****α** | 6 (4-8) | 5 (3-8) | 458 (281-753) | 213 (180-553)^#^ | 60 (45-94) | 28 (21-68)* |
| **IL-6** | 3 (3-3) | 3 (3-3) | 520 (319-703) | 300 (169-386)* | 68 (40-84) | 29 (18-49)* |
| **IL-8** | 6 (4-10) | 4 (3-7) | 590 (422-677) | 368 (266-540)* | 95 (58-109) | 47 (36-58)* |
| **IL-10** | 3 (3-5) | 3 (3-6) | 268 (220-648) | 962 (498-1208)* | 40 (38-81) | 116 (74-190)* |

TNF: tumor necrosis factor; IL: interleukin. Data are presented as median (interquartile range) of 12 subjects per group. ^#^ *p* = 0.05 - 0.10 vs. control, * *p* < 0.05 vs. control (Mann-Whitney U-tests). These data were published previously [1].

**

**

**Figure S1. Enriched pathways after LPS administration.** Significantly enriched pathways 4 hours after LPS administration compared with baseline (one hour before LPS administration). Threshold for significance was set at a Benjamini-Hochberg false discovery rate (FDR)-adjusted *p*-value of less than 0.1. Number of significantly increased/decreased metabolites within each pathway are indicated in parentheses.

**
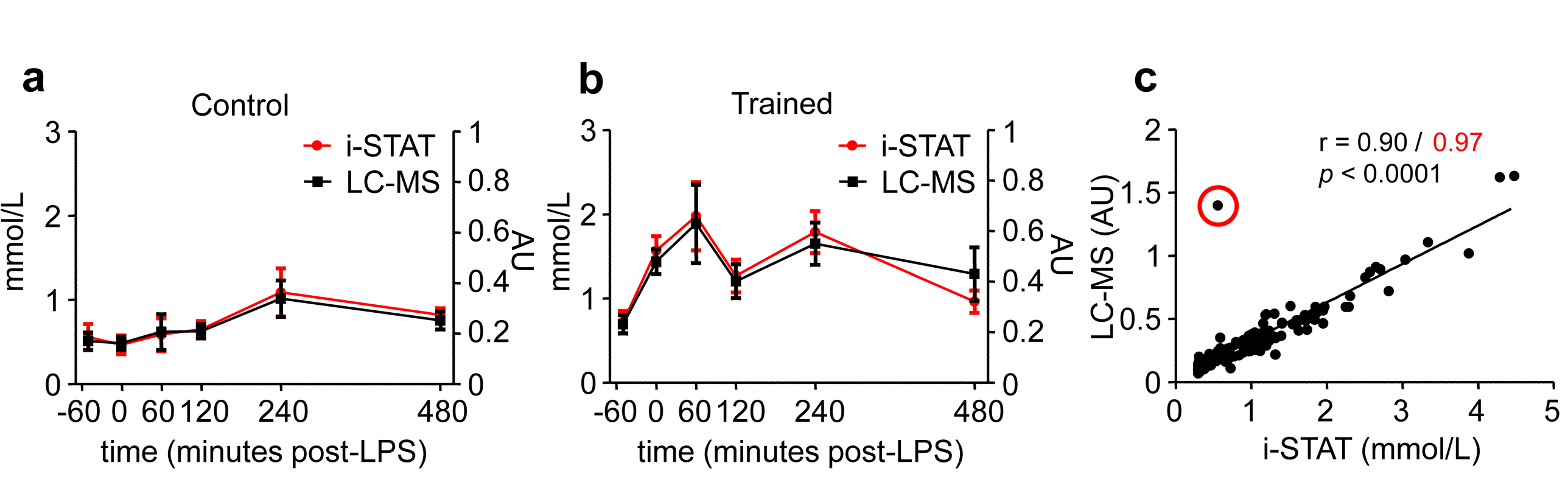
**

**Figure S2.** **Comparison of plasma lactate concentrations measured by LC-MS and a point-of-care analyzer. (a)** Plasma lactate concentrations over time in the control group, measured by a point-of-care blood analyzer (i-STAT) or by liquid chromatography – mass spectrometry (LC-MS); **(b)** Plasma lactate concentrations over time in the trained group, measured by i-STAT or LC-MS; **(c)** Correlation between plasma lactate concentrations measured by i-STAT or LC-MS. r and *p*-values were calculated using Pearson correlation. The r value in red was calculated after exclusion of the outlier indicated by the red circle. The i-STAT data presented in this figure were published previously [1]. Data in panels A-B are depicted as mean ± standard error of the mean (SEM) of 12 subjects per group.

**References**

1. Kox, M.; van Eijk, L.T.; Zwaag, J.; van den Wildenberg, J.; Sweep, F.C.; van der Hoeven, J.G.; Pickkers, P. Voluntary activation of the sympathetic nervous system and attenuation of the innate immune response in humans. *Proc Natl Acad Sci U S A* **2014**, *111*, 7379-7384, doi:10.1073/pnas.1322174111.
